# Policies and Management Interventions to Enhance Health and Care Workforce Capacity for Addressing the COVID-19 Pandemic: Protocol for a Living Systematic Review

**DOI:** 10.2196/50306

**Published:** 2023-10-05

**Authors:** Ana Paula Cavalcante de Oliveira, Mariana Lopes Galante, Leila Senna Maia, Isabel Craveiro, Alessandra Pereira da Silva, Ines Fronteira, Raphael Chança, Paulo Ferrinho, Mario Dal Poz

**Affiliations:** 1 Instituto de Medicina Social Universidade do Estado do Rio de Janeiro Rio de Janeiro Brazil; 2 Global Health and Tropical Medicine Instituto de Higiene e Medicina Tropical NOVA University of Lisbon Lisbon Portugal; 3 National School of Public Health Public Health Research Centre, Comprehensive Health Research Center NOVA University of Lisbon Lisbon Portugal; 4 Instituto Nacional de Cancer Ministério da Saúde Rio de Janeiro Brazil

**Keywords:** COVID-19, health and care workforce policy, health and care workforce capacity, management interventions, SARS-CoV-2, pandemic

## Abstract

**Background:**

Countries and health systems have had to make challenging resource allocation and capacity-building decisions to promote proper patient care and ensure health and care workers’ safety and well-being, so that they can effectively address the present COVID-19 pandemic as well as upcoming public health problems and natural catastrophes. As innovations are already in place and updated evidence is published daily, more information is required to inform the development and implementation of policies and interventions to improve health and care workforce capacity to address the COVID-19 pandemic response.

**Objective:**

The objective of this protocol review is to identify countries’ range of experiences with policies and management interventions that can improve health and care workers’ capacity to address the COVID-19 pandemic response and synthesize evidence on the effectiveness of the interventions.

**Methods:**

We will conduct a living systematic review of quantitative, qualitative, and mixed methods studies and gray literature (technical and political documents) published in English, French, Hindi, Portuguese, Italian, and Spanish between January 1, 2000, and March 1, 2022. The databases to be searched are MEDLINE (PubMed), Embase, SCOPUS, and Latin American and Caribbean Health Sciences Literature. In addition, the World Health Organization’s COVID-19 Research Database and the websites of international organizations (International Labour Organization, Economic Co-operation and Development, and The Health System Response Monitor) will be searched for unpublished studies and gray literature. Data will be extracted from the selected documents using an electronic form adapted from the Joanna Briggs Institute quantitative and qualitative tools for data extraction. A convergent integrated approach to synthesis and integration will be used. The risk of bias will be assessed with Joanna Briggs Institute critical appraisal tools, and the certainty of the evidence in the presented outcomes will be assessed with the Grading of Recommendations, Assessment, Development and Evaluation.

**Results:**

The database and gray literature search retrieved 3378 documents. Data are being analyzed by 2 independent reviewers. The study is expected to be published by the end of 2023 in a peer-reviewed journal.

**Conclusions:**

This review will allow us to identify and describe the policies and strategies implemented by countries and their effectiveness, as well as identify gaps in the evidence.

**Trial Registration:**

PROSPERO CRD42022327041; https://www.crd.york.ac.uk/prospero/display_record.php?RecordID=327041

**International Registered Report Identifier (IRRID):**

RR1-10.2196/50306

## Introduction

Health systems worldwide have been challenged by the COVID-19 pandemic, which affected about 670 million people as of May 2023 [1]. The need for the health and care workforce (HCWF) to be adaptable and flexible in the face of constant change was emphasized, as almost all countries affected by the pandemic faced a surge in reported cases and disruptions of essential health services [2].

The pandemic resulted in a high incidence of SARS-CoV-2 infections and deaths among health and care workers (HCWs); increased workload (accentuated by absenteeism and quarantine); and exposure to work-related health hazards and their consequences, including fatigue, psychosocial stress, despair, violence, and a shortage of personal protective equipment, among others [3,4]. Moreover, it highlighted preexisting weaknesses in health and care systems and services, such as a shortage of HCWs, as well as other sorts of imbalances that are long-standing challenges observed in almost every country [5].

A recent study concerning HCWs’ perceptions of government responses, support, and the impact of their measures on public and private health sectors identified dissatisfaction in most cases [6]. Particularly, they were discontent with the lack of health system organization; unequal distribution of support across health facilities; and the perception of government policies as disorganized, confused, and even contradictory [6]. Countries and health systems had to make challenging resource allocation and capacity-building decisions to promote proper patient care, ensure staff safety and well-being, and innovate to address the pandemic as well as upcoming public health problems and natural catastrophes. The widespread and far-reaching repercussions of this pandemic and other recent crises signal an urgent appeal to leaders and health and care systems around the world to be better prepared. Specifically, there should be clear and comprehensive disaster response management strategies [7] and a strong public health system, with HCWs who are fit for the purpose, well supported, better protected, and provided with decent working conditions.

The World Health Organization (WHO) has made an effort to synthesize high-quality, timely evidence and contribute guidance to inform the decision-making process. Their aim is to “assist health managers and policy-makers at national, subnational, and facility levels in designing, managing and preserving the workforce necessary to manage the COVID-19 pandemic and maintain essential health services” [8]. With the development of the epidemic, innovations are already in place and updated evidence is published frequently; however, more information is required to inform the development and implementation of policies and interventions.

Hence, we intend to conduct a living systematic review (LSR) to identify the range of experiences across countries with various policies and management interventions that can improve HCWs’ capacity to address the COVID-19 pandemic response, gather and synthesize evidence on the effectiveness of the interventions, and identify gaps and maintain the currency of the evidence.

## Methods

### Study Design

An LSR is a systematic review that is regularly updated, adding pertinent new information as it becomes available and serving as a crucial connection between health research findings and evidence-based health care decision-making [9].

With the current LSR (registered at PROSPERO; CRD42022327041 [10]), we intend to address the following review question: What strategies and policies have been adopted by countries to improve HWCs’ capacity to address the COVID-19 pandemic and how effective have they been?

An LSR is particularly appropriate to address this review question given that the issue under analysis is a priority for decision-making, the level of uncertainty in the existing evidence is high, and emerging evidence might impact the conclusions of a traditional systematic review. Most methodological guidelines still do not address the specificities of LSRs. Therefore, the proposed LSR will be conducted by adopting and adapting the Joanna Briggs Institute (JBI) methodology for mixed methods systematic reviews [10].

### Eligibility Criteria

The eligibility criteria for this LSR are as follows:

- Types of study: This review will include quantitative, qualitative, and mixed methods studies and gray literature (technical and political documents). Quantitative studies will include observational (eg, case-control, cohort, and cross-sectional), experimental, and quasi-experimental studies. Advocacy materials, opinion letters, news releases, editorials, and opinion documents will be excluded from the review but can be used to discuss the findings.
- Interventions: This review will consider countries’ range of policies and management interventions implemented to improve HCWF capacity to address the COVID-19 pandemic response, including the following domains: supporting and protecting health workers; strengthening and optimizing health workforce teams; and increasing capacity, strategic health worker deployment, and system-level human resources for health interventions as proposed by the WHO interim guide [8].
- Participants: The review will consider studies that address policies or management interventions implemented by countries regarding HCWs, hereby understood as all health-related occupations in the health and social sectors of employment, those working in health facilities offering all levels of care, and various employment settings.
- Context: This review will consider studies that investigate the range of interventions in different countries, whether implemented at supranational, national, or state levels.

### Search Strategy

The search strategy will aim to identify both published and unpublished studies. Search terms (preferably Medical Subject Headings [MeSH]) will be used to search the databases. To search relevant documents in databases that do not index using MeSH terms, we will use other thesaurus databases (eg, Descritores em Ciências da Saúde and Emtree) and the entry terms for each relevant keyword combined using the Boolean commands AND, OR, and * to capture various terms of the same word or expression (Multimedia Appendix 1).

The search of all data sources will be conducted by 1 researcher. After this process is complete, a second researcher will select a random sample of the databases to be used in a quality check. The reference list of all studies selected for critical appraisal will be screened for additional studies. Researchers from ongoing studies might be contacted to request preliminary or unpublished data to complete the publicly available data.

Studies published in English, French, Hindi, Portuguese, Italian, and Spanish will be included. The initial literature review will include studies published from January 1, 2020, to March 1, 2022*.* Since this systematic review is an LSR, monthly updates will be run for electronic databases. Studies identified will be incorporated in the review or noted in the “What’s new” section and included in the updates. Gray literature and international organizations or databases on workforce will be updated every 3 months.

The databases to be searched are MEDLINE (PubMed), Embase, SCOPUS, and Latin American and Caribbean Health Sciences Literature. Sources of unpublished studies and gray literature to be searched are the WHO’s COVID-19 Research Database [11] and the websites of international organizations: International Labour Organization [12], Economic Co-operation and Development [13], and The Health System Response Monitor [14].

### Study Selection

We will use EndNote Web (Clarivate) to collect, organize, and manage references retrieved from the searches of the different databases.

Data on eligibility will be collected through Rayyan (Rayyan Systems Inc.) [15]. Inclusion and exclusion criteria will be first applied to the abstracts of the retrieved documents. In case no abstract is available, the criteria will be applied either to the executive summary or the introduction of the documents. Titles and abstracts of the initial searches will be screened, and if eligibility criteria are met, the documents will be selected for full-text analysis. An initial pilot test will be performed in this phase (“Title+Abstract phase”) by reviewers until a sufficient level of agreement is reached. After that, the remaining publications will be divided between the reviewers. In this second phase, the eligibility criteria will again be applied to the full text independently by 2 researchers. If the text meets the criteria, we will then extract relevant data from the document to answer the research question. The reviewers are expected to collaborate whenever questions or doubts arise, and a third reviewer will be consulted in case of disagreement. κ statistics, sensibility, and sensitivity will be computed to assess interreviewer agreement and the quality of this process [16]. The inclusion of studies will be described according PRISMA (Preferred Reporting Items for Systematic Reviews and Meta-Analyses) recommendations, including the number of documents retrieved; identified as duplicates; screened for abstract; excluded and the reason for exclusion; and included during full-text analysis and the number of documents for data extraction [17].

### Risk of Bias and Quality Assessment

If the inclusion criteria are met, the documents will be selected for full-text analysis. In this phase, the eligibility criteria (as well as the exclusion criteria) will be again applied, and if they are met, we will then use the JBI critical appraisal tools (CATs) to assess the risk of bias in the included studies [18]. Quantitative and qualitative studies selected for retrieval will be assessed by 2 independent reviewers for methodological validity prior to inclusion in the review using the standardized critical appraisal instrument from JBI SUMARI; these instruments are designed to be study specific and facilitate cross-study comparisons. We have selected the following appraisal tools: randomized controlled trials, cohort studies, case-control studies, economic evaluations, qualitative studies, as well as text and opinion.

According to the assessment, we will then categorize the study into low, medium, and high quality, as follows:

1. Each item in the CAT has 4 options of answer (yes, no, unclear, or not applicable), to which we will assign the following points: yes=2 points, no=0 points, unclear=1 point, and not applicable=missing value and will not be considered for scoring.
2. We will compute a score per study by adding the points of each item.
3. For each CAT, the maximum score will be computed by multiplying the total number of items by the maximum score in each item (as detailed in Table 1.
4. Low quality is set at ≤quartile 2 of the maximum score, medium quality is set between quartile 2 + 1 and quartile 3, and high quality is set as ≥quartile 3 + 1. In case an integer value was not obtained, the next integer value was set as a cut-off.

**Table 1:** Critical appraisal tool (CAT) scores (adapted from Fronteira et al [19]).

CAT	Items	Maximum score	Quality and score range		
			Low	Medium	High
Checklist for Analytical Cross-Sectional Studies	8	16	≤8	9-12	≥13
Checklist for Case Control Studies	10	20	≤10	11-15	≥16
Checklist for Cohort Studies	11	22	≤11	12-17	≥18
Checklist for Prevalence Studies	9	18	≤9	10-14	≥15
Checklist for Qualitative Research	10	20	≤10	11-15	≥16
Checklist for Quasi-Experimental Studies	9	18	≤10	10-14	≥15
Checklist for Randomized Controlled Trials	13	26	≤13	14-20	≥21

The level of bias will be considered in the synthesis of the results on the evidence found. The CATs will be applied by 2 reviewers. The critical appraisal results will be reported in a narrative form and in a table. All studies, regardless of the results of their methodological quality, will undergo data extraction and synthesis (when possible).

The quality of the evidence supporting study findings regarding the outcomes of the policies and management interventions implemented by countries will be analyzed using the GRADE (Grading of Recommendations, Assessment, Development and Evaluation) approach developed by the GRADEpro Working Group [20].

### Data Extraction and Synthesis

Data will be extracted from the selected documents, including bibliographic information (author, year, location, and setting), study design type, interventions type, country, level or location, sector and actors involved, participant profession, sample size, delivery model, outcomes, and implementation context according to the data extraction instrument (Multimedia Appendix 2). The reviewers will use electronic forms adapted from the quantitative and qualitative tools for data extraction from the JBI Manual of Evidence Syntheses [21].

Study data will be collected and managed using REDCAP (Research Electronic Data Capture; Vanderbilt University) tools hosted at Universidade Estadual do Rio de Janeiro, Instituto de Medicina Social; the REDCAP form was specifically developed for the study design (quantitative or qualitative) [22,23]. The quantitative data will then be converted into “qualitized data.” This will involve transformation into textual descriptions or narrative interpretation of the quantitative results in a way that answers the review questions.

The primary outcomes to be considered are HCWF availability, distribution, performance, efficiency, productivity, retention, protection, working conditions, and satisfaction. Additional outcomes cover absenteeism, deaths, infection, cases of violence and harassment, turnover, intention to leave, workplace hazards, financial protection, service delivery disruptions or disrupted access to essential health services (continuity of treatment of chronic diseases), and coverage. Furthermore, additional outcomes not mentioned here can be added during the LSR.

This review will follow a convergent integrated approach according to the JBI methodology for mixed methods systematic review using JBI SUMARI that “refers to a process of combining extracted data from quantitative studies (including data from the quantitative component of mixed methods studies) and qualitative studies (including data from the qualitative component of mixed methods studies) and involves data transformation” [24]. It is recommended that quantitative data be “qualitized,” as codifying quantitative data is less error-prone than attributing numerical values to qualitative data [10] by assembling the qualitized data with the qualitative data. Assembled data are categorized and pooled together based on similarity in meaning to produce a set of integrated findings in the form of a line of action statements. This transformation leads to the creation of qualitative categories, which can be compared with other qualitative observations to identify patterns or discrepancies, thus extracting additional information from the quantitative data and validating its interpretation.

We will be grouping the findings in each specific policy and intervention domain per type of intervention and outcome. For each outcome, we will summarize the study type and method in selected studies, intervention context, population, outcomes, and risk-of-bias assessment results. We will discuss the body of evidence for each policy and intervention domain, the type of intervention and outcome, and the overall quality of the evidence by using the risk assessment results and GRADE recommendation system.

The type of quantitative synthesis of data will be determined by evidence for a prespecified comparison, the completeness of the reported outcome, differences in the effect measures, bias in the evidence, clinical and methodological diversity, and statistical heterogeneity.

## Results

The database and gray literature search retrieved 3378 documents. Data are being analyzed by 2 independent reviewers. The results are expected to be published in a peer-reviewed journal by the end of 2023.

## Discussion

### Expected Findings

The COVID-19 pandemic has revealed significant vulnerabilities and challenges within global health and care systems, particularly in terms of HCWF capacity and resilience. Policies and management interventions implemented to improve the HCWF’s capacity to address the COVID-19 pandemic response have been discussed in different studies [25-30]. For example, one study suggested methods to measure and improve the resilience and preparedness of countries to face the pandemic through timely and responsive strategies for crisis management to mitigate negative impacts on public health, economies, and societies [30]. It has also been identified that failures in the management of the pandemic with regard to ensuring workers’ well-being can result in exacerbated inequality and highlight the importance of politics in countries, where political authority also shapes decision-making processes [29]. In addition, sound leadership and ownership lead to the successful execution of initiatives and overall preparedness. The adaptability of health services plays a crucial role in achieving positive outcomes [31]. However, only a few studies have mentioned the outcomes of the interventions and addressed it in a systematic manner [27].

To the best of our knowledge, this is the first LSR that represents a comprehensive examination of policies and management interventions across 4 levels—individual, management, organizational, and system—in the context of pandemics. The study design; the incorporation of scientific, technical, and political documents; and the use of a framework are strengths of this review, as they offer a broad perspective on the identities of the interventions implemented (encompassing information that, by its nature, does not make it into scientific documents). This presents an objective view of the topic while minimizing biases by following established procedures and reporting guidelines. Other strengths include the robust search strategy, which includes published and unpublished literature in 6 languages across 5 databases and the websites of international organization. In addition, we will report the percentage of agreement between reviewers.

On the contrary, the limitations of this systematic review may include publication bias, as studies with favorable outcomes are more likely to be published, resulting in an overrepresentation of effective interventions. In the context of a pandemic, policies and strategies to enhance the HCWF are also rapidly evolving. In addition, the impact of the pandemic may evolve over time, making it difficult to assess the long-term effects and sustainability of the proposed policies and strategies.

### Conclusions

This review can shed light on measures to improve the HCWF; the results can guide policy makers in developing evidence-informed policies and strategies that optimize the HCW response to future pandemics. Findings can inform future policy decisions and resource allocation. In addition, by conducting an LSR that incorporates the latest evidence, health care systems can respond more swiftly to the changing demands of HCWs. Furthermore, identifying gaps in knowledge can contribute to the development of a research agenda to inform the development of policies and strategies related to the HCWF.

## Appendix

Multimedia Appendix 1 Search strategy.

Multimedia Appendix 2 Data extraction instrument.

## Abbreviations

CAT: critical appraisal tool
HCW: health and care worker
HCWF: health and care workforce
HSRM: The Health System Response Monitor
JBI: Joanna Briggs Institute
LSR: Living Systematic Review
MeSH: Medical Subject Headings
WHO: World Health Organization

## Data Availability

The database of the living systematic review is available upon request from the authors.
