# Supplementary material for: Policies and Management Interventions to Enhance Health and Care Workforce Capacity for Addressing the COVID-19 Pandemic: Protocol for a Living Systematic Review
Source: JMIR Res Protoc. 2023 Oct 5;12:e50306. doi: 10.2196/50306 (PMC10587809; doi:10.2196/50306)
Supplement: Multimedia Appendix 1 [file resprot_v12i1e50306_app1.docx]

## Multimedia Appendix 1

To identify the search terms and expressions to search sites and databases, we have identified relevant keywords from the research questions (Health workforce; Policy and management interventions; COVID-19) and referenced articles. To identify the search terms, the controlled health vocabularies DeCs (Descriptors in Health Sciences), MeSH (Medical Subject Headings) and Emtree (Embase Subject Headings) were consulted. Relevant words not captured in the keyword search were included as free terms. Only literature available in English, French, Hindi, Portuguese, Italian and Spanish and published after 2020 will be considered.

The search strategy in the database used descriptors, and entry terms, with Boolean commands AND, OR, *, and NOT for various terminations of the same word/ expression. The identification and organization of terms were carried out according to the peculiarities of each database. The search strategy developed was tested in the US National Library of Medicine National Institutes of Health (PUBMED), EMBASE, SCOPUS, Literatura Latino-Americana e do Caribe em Ciências da Saúde via BVS (BVS/LILACS) and WHO COVID-19 databases in March 2022, as presented.

| DATABASE | Search Strategies | Records retrieved |
| --- | --- | --- |
| PUBMED | (((Health Workforce[mj] OR Workforce*[tiab] OR Health Manpower[tiab] OR Health Personnel[mj] OR Health Personnel*[tiab] OR Health Care Provider*[tiab] OR Healthcare Provider*[tiab] OR Health Care Worker*[tiab] OR Healthcare Worker*[tiab] OR Health Care Professional*[tiab] OR Healthcare Professional*[tiab] OR Human Resources for Health[tiab] OR Caregivers[mj] OR Caregiver*[tiab] OR Licensed Practical Nurses[mj] OR Nursing Staff[mj] OR Nurses[mj] OR Nurse*[tiab] OR Nursing Personnel*[tiab] OR Nursing Staff*[tiab] OR Professional Nurse*[tiab] OR Nursing Associate*[tiab] OR Nursing Professional*[tiab] OR Nursing Assistant*[tiab] OR Auxiliary Nurse*[tiab] OR Nursing Auxiliar*[tiab] OR Licensed Practical Nurse*[tiab] OR Nursing Team*[tiab] OR Dentist*[tiab] OR Doctor*[tiab] OR Physicians[mj] OR Physician*[tiab] OR Pharmacist*[tiab] OR Physiotherapist*[tiab] OR Midwive*[tiab] OR Community Health Worker*[tiab] OR Community-Based Provider*[tiab] OR Laboratory Staff*[tiab] OR Paramedical Staff*[tiab] OR Paramedical Personnel*[tiab]) AND (Health Policy[mj] OR Health Planning[mj] OR Health Planning[ti] OR Polic*[ti] OR Intervention*[ti] OR Strateg*[ti] OR Measure*[ti] OR Shortage*[ti] OR Pandemic Response[ti])) AND (COVID-19[mj] OR SARS-CoV-2[mj] OR Severe Acute Respiratory Syndrome Coronavirus 2[tiab] OR Coronavirus Disease 2019[tiab] OR 2019 Novel Coronavirus[tiab] OR 2019 New Coronavirus[tiab] OR Wuhan Coronavirus[tiab] OR COVID-19[tiab] OR SARS-CoV-2[tiab] OR 2019-nCoV[tiab] OR HCoV-19[tiab] OR nCoV-2019[tiab] OR Novel Coronavirus 2019-nCoV[tiab] OR Alpha Variant[tiab] OR Beta Variant[tiab] OR Gamma Variant[tiab] OR Delta Variant[tiab] OR Delta Plus Variant[tiab] OR Omicron Variant[tiab] OR Lambda Variant[tiab])) NOT (Letter*[ti] OR Editor*[ti] OR Release*[ti]) AND (English[lang] OR Portuguese[lang] OR Spanish[lang] OR French[lang] OR Italian[lang] OR Hindi[lang]) AND ("2020/01/01"[PDAT] : "2022/03/01"[PDAT]) | **1727** |
| EMBASE | ('health workforce'/mj OR 'health care labour force':ti,ab OR 'health care manpower':ti,ab OR 'health care work force':ti,ab OR 'health care workforce':ti,ab OR 'health labor force':ti,ab OR 'health labour force':ti,ab OR 'health manpower':ti,ab OR 'health work force':ti,ab OR 'health workforce':ti,ab OR 'healthcare labor force':ti,ab OR 'healthcare labour force':ti,ab OR 'healthcare manpower':ti,ab OR 'healthcare work force':ti,ab OR 'healthcare workforce':ti,ab OR 'health care labor force':ti,ab OR 'health care personnel'/mj OR 'health care personnel*':ti,ab OR 'health care practitioner*':ti,ab OR 'health care professional*':ti,ab OR 'health care provider*':ti,ab OR 'health care worker*':ti,ab OR 'health personnel*':ti,ab OR 'health worker*':ti,ab OR 'healthcare personnel':ti,ab OR 'healthcare practitioner':ti,ab OR 'healthcare professional':ti,ab OR 'healthcare provider':ti,ab OR 'healthcare worker*':ti,ab OR 'caregiver'/mj OR 'caregiver*':ti,ab OR 'human resources for health':ti,ab OR 'licensed practical nurse'/mj OR 'licensed practical nurse*':ti,ab OR 'licensed vocational nurse':ti,ab OR 'nursing staff'/mj OR 'hospital nursing staff':ti,ab OR 'nurse staffing':ti,ab OR 'nursing manpower':ti,ab OR 'nursing personnel':ti,ab OR 'nursing staff':ti,ab OR 'nurse'/mj OR 'nurse*':ti,ab OR 'nursing associate*':ti,ab OR 'nursing assistant'/mj OR 'nursing assistant*':ti,ab OR 'auxiliary nurse*':ti,ab OR 'nursing auxiliar*':ti,ab OR 'team nursing'/mj OR 'team nursing':ti,ab OR 'dentist'/mj OR 'dentist*':ti,ab OR 'physician'/mj OR 'doctor*':ti,ab OR 'physician*':ti,ab OR 'private physician':ti,ab OR 'pharmacist'/mj OR 'pharmacist*':ti,ab OR 'physiotherapist'/mj OR 'physical therapist*':ti,ab OR 'physiotherapist*':ti,ab OR 'midwife'/mj OR 'midwife':ti,ab OR 'midwifery':ti,ab OR 'midwives':ti,ab OR 'health auxiliary'/mj OR 'auxiliary health worker':ti,ab OR 'community health worker*':ti,ab OR 'medical auxiliary':ti,ab OR 'community-based provider*':ti,ab OR 'laboratory staff*':ti,ab OR 'paramedical personnel'/mj OR 'healthcare assistant':ti,ab OR 'healthcare support worker':ti,ab OR 'para medical personnel':ti,ab OR 'paramedical personnel':ti,ab OR 'paramedical professional':ti,ab OR 'paramedical staff':ti,ab) AND ('health care policy'/mj OR 'health care policy':ti OR 'health policy':ti OR 'healthcare policy':ti OR 'health care planning'/mj OR 'community health planning':ti OR 'health and welfare planning':ti OR 'health care planning':ti OR 'health planning':ti OR 'health planning technical assistance':ti OR 'health systems plans':ti OR 'healthcare and welfare planning':ti OR 'healthcare planning':ti OR polic*:ti OR intervention*:ti OR strateg*:ti OR measure*:ti OR shortage*:ti OR 'pandemic response*':ti) AND ('coronavirus disease 2019'/exp OR '2019 novel coronavirus':ti,ab OR '2019-ncov':ti,ab OR 'covid 19':ti,ab OR 'covid 2019':ti,ab OR 'covid-19':ti,ab OR 'sars coronavirus 2':ti,ab OR 'sars-cov-2':ti,ab OR 'wuhan coronavirus':ti,ab OR 'coronavirus disease 2019':ti,ab OR 'coronavirus infection 2019':ti,ab OR 'ncov 2019':ti,ab OR 'new coronavirus':ti,ab OR 'novel coronavirus 2019':ti,ab OR 'novel coronavirus':ti,ab OR 'severe acute respiratory syndrome 2':ti,ab OR 'Alpha Variant':ti,ab OR 'Beta Variant':ti,ab OR 'Gama Variant':ti,ab OR 'Delta Variant':ti,ab OR 'Delta Plus Variant':ti,ab OR 'Omicron Variant':ti,ab OR 'Lambda Variant':ti,ab) NOT (letter*:ti,ab,kw OR editorial*:ti,ab,kw OR release*:ti,ab,kw) AND ([english]/lim OR [french]/lim OR [hindi]/lim OR [italian]/lim OR [portuguese]/lim OR [spanish]/lim) AND [01-01-2020]/sd NOT [01-03-2022]/sd AND [embase]/lim NOT ([embase]/lim AND [medline]/lim) | **521** |
| SCOPUS | TITLE("Health Workforce" OR "Health Workforces" OR "Health Manpower" OR "Health Personnel" OR "Health Personnels" OR "Health Care Providers" OR "Healthcare Providers" OR "Health Care Workers" OR "Healthcare Workers" OR "Health Care Professionals" OR "Healthcare Professionals" OR Caregiver* OR "Licensed Practical Nurses" OR "Nursing Staff" OR Nurse* OR "Nursing Personnel" OR "Professional Nurses" OR "Nursing Associate" OR "Nursing Professionals" OR "Nursing Assistant" OR "Auxiliary Nurses" OR "Nursing Auxiliary" OR "Licensed Practical Nurses" OR "Nursing Team" OR Dentists OR Doctors OR Physicians OR Pharmacists OR Physiotherapists OR Midwives OR "Community Health Workers" OR "Community-Based Providers" OR "Laboratory Staff" OR "Paramedical Staff" OR "Paramedical Personnel") AND TITLE("Health Policy" OR "Health Planning" OR Polic* OR Intervention* OR Strateg* OR Measure* OR Shortage* OR "Pandemic Response") AND ALL(COVID-19 OR SARS-CoV-2 OR "Severe Acute Respiratory Syndrome Coronavirus 2" OR "Coronavirus Disease 2019" OR "2019 Novel Coronavirus" OR "2019 New Coronavirus" OR "Wuhan Coronavirus" OR 2019-nCoV OR HCoV-19 OR nCoV-2019 OR "Novel Coronavirus 2019-nCoV" OR "Alpha Variant" OR "Beta Variant" OR "Gamma Variant" OR "Delta Variant" OR "Delta Plus Variant" OR "Omicron Variant" OR "Lambda Variant") AND (LIMIT-TO(DOCTYPE, "ar")) AND (LIMIT-TO(LANGUAGE, "English") OR LIMIT-TO(LANGUAGE, "Spanish") OR LIMIT-TO(LANGUAGE, "French") OR LIMIT-TO(LANGUAGE, "Portuguese")) OR LIMIT-TO(LANGUAGE, "Italian")) OR LIMIT-TO(LANGUAGE, "Hindi")) AND (LIMIT-TO(PUBYEAR, 2022) OR LIMIT-TO(PUBYEAR, 2021) OR LIMIT-TO(PUBYEAR, 2020)) | **454** |
| BVS/LILACS | (covid-19 OR sars-cov-2 OR "Severe Acute Respiratory Syndrome Coronavirus 2" OR "Coronavirus Disease 2019" OR "2019 Novel Coronavirus" OR "2019 New Coronavirus" OR "Wuhan Coronavirus" OR 2019-ncov OR hcov-19 OR ncov-2019 OR "Novel Coronavirus 2019-nCoV" OR "Sindrome Respiratória Aguda Grave 2" OR "Novo Coronavirus" OR "Alpha Variant" OR "Beta Variant" OR "Gamma Variant" OR "Delta Variant" OR "Delta Plus Variant" OR "Omicron Variant" OR "Lambda Variant") AND ("Health Workforce" OR "Health Workforces" OR "Health Manpower" OR "Health Personnel" OR "Health Personnels" OR "Health Care Providers" OR "Healthcare Providers" OR "Health Care Workers" OR "Healthcare Workers" OR "Health Care Professionals" OR "Healthcare Professionals" OR caregiver* OR "Licensed Practical Nurses" OR "Nursing Staff" OR Nurse* OR "Nursing Personnel" OR "Professional Nurses" OR "Nursing Associate" OR "Nursing Professionals" OR "Nursing Assistant" OR "Auxiliary Nurses" OR "Nursing Auxiliary" OR "Licensed Practical Nurses" OR "Nursing Team" OR Dentists OR Doctors OR Physicians OR Pharmacists OR Physiotherapists OR Midwives OR "Community Health Workers" OR "Community-Based Providers" OR "Laboratory Staff" OR "Paramedical Staff" OR "Paramedical Personnel" OR "Força de trabalho em saúde" OR "Recursos humanos em saúde" OR "Pessoal de saúde" OR "Provedores de saúde" OR "Trabalhadores em saúde" OR "Serviços de saúde Profissionais" OR "Profissionais de saúde" OR Cuidador* OR "Enfermeiros práticos licenciados" OR "Equipe de enfermagem" OR Enfermeira* OR "Pessoal de enfermagem" OR "Enfermeiros profissionais" OR "Profissionais de enfermagem" OR "Assistente de enfermagem" OR "Auxiliares de enfermagem" OR dentista* OR médico* OR farmacêutico* OR fisioterapeuta* OR parteira* OR "Trabalhadores comunitários de saúde" OR "Provedores de base comunitária" OR "Equipe de laboratório" OR "Pessoal paramédico" OR "Personal de salud" OR "Recursos humanos en salud" OR "Proveedores de salud" OR "Trabajadores de la salud" OR "Servicios profesionales de salud" OR "Profesionales de la salud" OR "Enfermeras prácticas con licencia" OR "Personal de enfermería" OR Enfermera* OR "Personal de enfermería" OR "Profesionales de enfermería" OR "Auxiliar de enfermería" OR "Auxiliares de enfermería" OR partera* OR "Trabajadores de salud comunitarios" OR "Proveedores comunitarios" OR "Personal de laboratorio" OR paramédico*) AND (ti:("Health Policy" OR "Health Planning" OR Polic* OR Intervention* OR Strateg* OR Measure* OR Shortage* OR "Pandemic Response" OR "Políticas de Saúde" OR "Planejamento de Saúde" OR Politica* OR Estratégia* OR Intervenç* OR Medida* OR Escassez OR Baixa OR "Resposta a Pandemia" OR "Política de salud" OR "Planificación de salud" OR Intervención* OR Medida* OR Escasez OR Baja OR "Respuesta a pandemia")) AND (db:("LILACS")) AND (year_cluster:[2000 TO 2022]) | **145** |
| WHO COVID-19 | (tw:("Health Workforce" OR "Health Workforces" OR "Health Manpower" OR "Health Personnel" OR "Health Personnels" OR "Health Care Providers" OR "Healthcare Providers" OR "Health Care Workers" OR "Healthcare Workers" OR "Health Care Professionals" OR "Healthcare Professionals" OR caregiver* OR "Licensed Practical Nurses" OR "Nursing Staff" OR nurse* OR "Nursing Personnel" OR "Professional Nurses" OR "Nursing Associate" OR "Nursing Professionals" OR "Nursing Assistant" OR "Auxiliary Nurses" OR "Nursing Auxiliary" OR "Licensed Practical Nurses" OR "Nursing Team" OR dentists OR doctors OR physicians OR pharmacists OR physiotherapists OR midwives OR "Community Health Workers" OR "Community-Based Providers" OR "Laboratory Staff" OR "Paramedical Staff" OR "Paramedical Personnel")) AND (ti:("Health Policy" OR "Health Planning" OR polic* OR intervention* OR strateg* OR measure* OR shortage* OR "Pandemic Response") AND (covid-19 OR sars-cov-2 OR "Severe Acute Respiratory Syndrome Coronavirus 2" OR "Coronavirus Disease 2019" OR "2019 Novel Coronavirus" OR "2019 New Coronavirus" OR "Wuhan Coronavirus" OR 2019-ncov OR hcov-19 OR ncov-2019 OR "Novel Coronavirus 2019-nCoV" OR "Alpha Variant" OR "Beta Variant" OR "Gamma Variant" OR "Delta Variant" OR "Delta Plus Variant" OR "Omicron Variant" OR "Lambda Variant")) AND db:("GREY-COVIDWHO" OR "COVIDWHO") | **91** |
|  |  |  |
