# Supplementary material for: Policies and Management Interventions to Enhance Health and Care Workforce Capacity for Addressing the COVID-19 Pandemic: Protocol for a Living Systematic Review
Source: JMIR Res Protoc. 2023 Oct 5;12:e50306. doi: 10.2196/50306 (PMC10587809; doi:10.2196/50306)
Supplement: Multimedia Appendix 2 [file resprot_v12i1e50306_app2.docx]

**Multimedia Appendix 2**

**DATA EXTRACTION INSTRUMENT**

- **Evidence source details and characteristics**
  - Citation details: authors, title, journal, year
- **Context**
  - Name of country of intervention was implemented.
- **Intervention Characterization**
  - **Domain of policy management intervention**
    - Supporting and protecting health workers (individual level)
    - Strengthening and optimizing health workforce teams (management/health workforce)
    - Increasing capacity and strategic health worker deployment (Organizational environment)
    - System-level HRH interventions
  - Area of intervention (according to each domain above)

_______________________________________________________

- **Data extraction Qualitative**
  - Methodology
  - Setting
  - Phenomenon of interest
  - Intervention context (objective, sector and actors involved, implementation details, participants, duration)
  - Key findings related to review questions (when available)

- **Data extraction Quantitative**
  - Aims of the study
  - Study design
  - Setting
  - Intervention (objective, sector and actors involved, implementation details, participants)
  - Follow-up/study duration
  - Outcomes and outcome measurements
